# Brainstem auditory-evoked potentials in two meditative mental states

**DOI:** 10.4103/0973-6131.72628

**Published:** 2010

**Authors:** Sanjay Kumar, HR Nagendra, KV Naveen, NK Manjunath, Shirley Telles

**Affiliations:** Department of Yoga Research, Indian Council of Medical Research Center for Advanced Research in Yoga and Neurophysiology, SVYASA, Bangalore, India

**Keywords:** Brainstem auditory-evoked potential, *cancalata*, *dharana*, *dhyana*, *ekagrata*

## Abstract

**Context:**

Practicing mental repetition of “OM” has been shown to cause significant changes in the middle latency auditory-evoked potentials, which suggests that it facilitates the neural activity at the mesencephalic or diencephalic levels.

**Aims:**

The aim of the study was to study the brainstem auditory-evoked potentials (BAEP) in two meditation states based on consciousness, viz. *dharana*, and *dhyana*.

**Materials and Methods:**

Thirty subjects were selected, with ages ranging from 20 to 55 years (M=29.1; ±SD=6.5 years) who had a minimum of 6 months experience in meditating “OM”. Each subject was assessed in four sessions, i.e. two meditation and two control sessions. The two control sessions were: (i) *ekagrata*, i.e. single-topic lecture on meditation and (ii) *cancalata*, i.e. non-targeted thinking. The two meditation sessions were: (i) *dharana*, i.e. focusing on the symbol “OM” and (ii) *dhyana*, i.e. effortless single-thought state “OM”. All four sessions were recorded on four different days and consisted of three states, i.e. pre, during and post.

**Results:**

The present results showed that the wave V peak latency significantly increased in *cancalata*, *ekagrata* and *dharana*, but no change occurred during the *dhyana* session.

**Conclusions:**

These results suggested that information transmission along the auditory pathway is delayed during *cancalata*, *ekagrata* and *dharana*, but there is no change during *dhyana*. It may be said that auditory information transmission was delayed at the inferior collicular level as the wave V corresponds to the tectum.

## INTRODUCTION

The functions of the brain in meditation have been studied using different techniques. These include the electroencephalogram (EEG),[1] evoked potentials,[2] regional cerebral glucose utilization as well as, more recently, functional magnetic resonance imaging.[3] Among these methods, a specific technique is selected for each experiment as each of them have different spatial and temporal resolutions.[4]

Evoked potentials are used in meditation studies because a correlation between different evoked potential components and underlying neural generators is reasonably well worked out.[5] Apart from this, it appears that the cerebral cortex is actively involved in meditation.[6] Hence, one may expect corticoefferent gating with changes occurring at the subcortical relay centers.[7] For these reasons, there have been studies of short and midlatency auditory-evoked potentials during meditation. The studies on midlatency auditory-evoked potentials have most often shown changes in a component called the Na-wave, a negative wave occurring between 14 and 19 msec. The changes have been in the form of an increase in amplitude,[8] suggesting the requirement of more neurons. A decrease in latency has also been reported,[9] suggesting a decrease in time taken to transmit sensory information.

Studies on short latency auditory-evoked potentials have not shown such clear changes.[2] In that study, brainstem auditory evoked potentials (BAEP) were measured in five advanced practitioners of transcendental meditation (TM) to determine whether such responses would reflect an increase in perceptual acuity to auditory stimuli following meditation. The BAEP provide an objective physiological index of auditory function at a subcortical level. Repeated measures of the BAEP of TM practitioners were taken before and after a period of meditation and were compared with those of age-matched controls. Peak latencies as well as interwave latencies between major BAEP components were evaluated. No pre–post meditation differences for experimental subjects were observed at low-stimulus intensities (0–35 dB). At moderate intensities (40–50 dB), the latency of the inferior collicular wave (wave V) increased following meditation. However, at higher stimulus intensities (55–70 dB), the latency of this wave was slightly decreased. Comparison of the slopes and intercepts of stimulus intensity–latency functions indicate a possible effect of meditation on brainstem activity.[2] This study on short latency auditory-evoked potentials in TM meditation practitioners demonstrated that short latency auditory-evoked potential varies with stimulus characteristics.

More recently, we have attempted to understand meditation based on descriptions from an ancient yoga text. This is *Patanjali’s yoga sutras* (*circa* 900 BC).[10] Based on this description, meditation has been considered as two states, namely *dharana*, which is characterized by focusing on the object of meditation and *dhyana*, which is a defocused state of mental expansiveness. With this background, the present study was undertaken to determine whether short latency auditory-evoked potentials would change in normal subjects in meditation considered as both *dharana* and *dhyana* sessions on separate days.

## MATERIALS AND METHODS

### Subjects

Thirty subjects were selected in the age range between 20 and 55 years (group mean±SD, 29.1±6.5 years) recruited from a residential setup, Swami Vivekananda Yoga Research Foundation, Bangalore, in south India. This age range was selected as short latency does not vary within this age range in healthy individuals.[11] Only male subjects were selected because it has been demonstrated that short latency auditory-evoked potentials vary with the phases of the menstrual cycle.[12] All of them had normal health based on a routine case history and a clinical examination. Also, all of them had experience of practicing meditation for at least 30 min per day, 4 days in a week, for a minimum of 1 year. Their meditation practice was based on self-reporting of the meditators as well as (where possible) consultations with the meditation teacher (*guru*).

To assess the quality of the practice, visual analogue scale (VAS) was used at the end of each session.

All of them expressed their willingness to participate in the experiment. The project was approved by the Institution’s Ethics Committee. The study protocol was explained to the subjects and their signed informed consent was obtained.

Apart from their prior experience of “OM” meditation, they had undergone a 2-month orientation program in “OM” meditation under the guidance of an experienced meditation teacher.

The condition to exclude subjects were any health disorder, especially psychiatric or neurological disorders, auditory deficits assessed by checking the auditory threshold of each ear separately and any medication that alters the functions of the nervous system. None of the subjects had to be excluded for these reasons.

The order of the four sessions (i.e. two meditation sessions and two non-meditation control sessions) was randomized for each subject using a standard random number table.[13] This was done to prevent the influence of being exposed to the laboratory for the first time for example, from influencing the results among other reasons.

### Design

Each subject was assessed in four sessions, i.e. two meditation and two control sessions, to record BAEP. The two control sessions were: (i) *ekagrata*, i.e. single-topic lecture on meditation and (ii) *cancalata*, i.e. non-targeted thinking. The two meditation sessions were: (i) *dharana*, i.e. focusing on the symbol “OM” and (ii) *dhyana*, i.e. effortless single-thought state “OM.” All four sessions consisted of three states, i.e. “pre” (5 min), “during” (20 min) and “post” (5 min).

The assessments were made on four different days, not necessarily on consecutive days, but at the same time of the day (i.e., the self-as-control design). The allocation of the subjects to the four sessions was randomized using a standard random number table.[13] This was done to prevent the influence of being exposed to the laboratory for the first time from influencing the results.

### Assessments

BAEP were recorded using the Nicolet Bravo system (Nicolet Biomedicals, Madison, WI, USA). The amplifier settings were as follows: low-frequency filter 100 Hz, high-frequency filter 3 KHz, sensitivity 50 *µ*V, number of sweeps averaged 1,500, sweep width 10 ms, delay 0 ms. Binaural click stimuli, of alternating polarity, with 11.1 Hz frequency and 100 *µ*S duration, were delivered through acoustically shielded earphones (Amplivox, Oxfordshire, UK). The stimulus intensity was kept at 80 dB nHL. The rejection level was expressed as a percentage of the full-scale range of the analog-to-digital converter. This level was set at 90%. Silver chloride (Ag/AgCl) disc electrodes were placed on the scalp using a conductive water-soluble paste. The active electrode was at Cz according to the International 10–20 system[14] referenced to linked ear lobes, with the ground electrode on the forehead (FPz). All electrode impedances were kept below 5 KΩ throughout the session.

### Interventions

Throughout all sessions, the subjects kept their eyes closed and followed pre-recorded instructions. The instructions emphasized carrying out the practice slowly, with awareness and relaxation. The meditators who participated in the study underwent 1 month of orientation sessions, where they practiced two phases that formed a continuum in meditation (*dharana* and *dhyana*) as two separate states and two control states, i.e. *cancalata* or non-focused thinking and *ekagrata* or focusing without meditation and on more than one thought.

These states are described in the traditional texts, i.e. the *Patanjali’s yoga sutras* and *Bhagavad Gita*, stating that when awake and in the absence of a specific task, the mind is very distractible (*cancalata*), and has to be taken through the stages of “streamlining the thoughts” (concentration or *ekagrata*) before moving on to the states of meditation. These are: one-pointed concentration or *dharana* and a defocused, effortless single-thought state or *dhyana*.

In the *cancalata* session, the 20-min period consisted of “non-targeted thinking,” during which the subjects were asked to allow their thoughts to wander freely as they listened to a compiled audio CD consisting of brief periods of conversation and talks on multiple topics recorded from a local radio station transmission. In the *ekagrata* session, the 20-min period consisted of focusing on a single topic, which was listening to a lecture on meditation, with multiple, yet associated, thoughts. In the *dharana* session, the 20-min period consisted of focusing on the symbol “OM.” During this session, they were asked to focus on the meaning of the syllable, OM, which is used as a symbol for the entire universe because OM is considered to represent “that which sustains everything.”[15] In the *dhyana* session, the 20 min of the practice consisted of meditation with effortless absorption in the single-thought state of the object of meditation, i.e. “OM.”

For the two meditation sessions and the two control sessions, subjects were given guided instructions through separate recorded instructions for each session.

### Data extraction

For the BAEP, the peak latencies and peak amplitudes of all seven waves were calculated. Peak latency (msec) is defined as the time from stimulus onset to the point of maximum positive amplitude within the latency window. Peak amplitude (V) is defined as the voltage difference between a pre-stimulus baseline and the largest positive going peak within a given latency window.

### Data analysis

Statistical analysis was performed using SPSS (Version 10.0). The peak latencies and peak amplitudes of all seven waves were analyzed using repeated-measures analyses of variance (ANOVAs) and *post hoc* analyses with Bonferroni adjustment were performed to compare “pre” data with “during” and “post.”

The repeated measures ANOVAs were performed with two “within–subject” factors, i.e. Factor 1: Sessions; with four levels, viz. *cancalata*, *ekagrata*, *dharana* and *dhyana*, and Factor 2: States; with six levels, viz. *pre, during (D1 to D4) and post*. These repeated measures ANOVAs were carried out for the peak latency and peak amplitude of all levels.

This was followed by a *post hoc* analysis with Bonferroni adjustment for multiple comparisons between the mean values of different states (pre, during 1 to during 4 and post).

## RESULTS

The peak latency of wave V showed a significant difference between Sessions (*F*=3.894, for *df*=2.678, 77.651, *P*< 0.015, Huynh-Feldt epsilon=0.893) and between States (*F*=11.713, for *df*=4.181, 121.256, *P*< 0.001, Huynh-Feldt epsilon=0.836).

*post hoc* analysis with Bonferroni adjustment for each session (*cancalata*, *ekagrata*, *dharana* and *dhyana*) separately showed a significant increase in the latency of wave V during the *cancalata* session (pre versus during, i.e. D2; *P*=0.042), *ekagrata* session (pre versus during, i.e. D2; *P*=0.009, pre versus during, i.e. D3; *P*=0.026, pre versus during, i.e. D4; *P*=0.005 and pre versus post *P*=0.001) and following the *dharana* session (pre versus post; *P*=0.018).

The amplitude of wave V also showed a significant difference between Sessions (*F*=6.515, for *df*=2.692, 78.060, *P*< 0.001, Huynh-Feldt epsilon=0.897) and between States (*F*=8.574, for *df*=4.292, 124.456, *P*< 0.001, Huynh-Feldt epsilon=0.858).

*post hoc* analysis with Bonferroni adjustment for each session (*cancalata*, *ekagrata*, *dharana* and *dhyana*) separately showed no significant change in the peak amplitude of wave V (*P*>0.05). Also, there were no significant change in the other waves (*P*>0.05).

Hence, the changes in wave V peak latency alone are presented in Table 1.

**Table 1:** Latency of wave V brainstem auditory-evoked potentials (BAEP) in four sessions

Sessions	States					

	Pre	During 1	During 2	During 3	During 4	Post
*Cancalata*	5.78±0.18	5.82±0.18	5.84**±0.17	5.84±0.20	5.84±0.18	5.82±0.17
*Ekagrata*	5.76±0.19	5.83±0.18	5.83**±0.18	5.83*±0.17	5.87±0.19	5.85***±0.18
*Dharana*	5.75±0.20	5.80±0.19	5.80±0.19	5.78±0.21	5.80±0.21	5.82**±0.18
*Dhyana*	5.79±0.18	5.81±0.19	5.82±0.19	5.81±0.18	5.81±0.20	5.82±0.18

* *P*<0.05

** *P*<0.01

*** *P*<0.001; RM ANOVA with Bonferroni adjustment compared state with pre.

## DISCUSSION

In the present study, normal healthy volunteers who were experienced in practicing meditation on the syllable “OM” were assessed in two meditation (i.e., *dharana* and *dhyana*) and two control sessions (i.e., *cancalata* and *ekagrata* sessions). BAEP were recorded throughout all four sessions. There was a significant increase in the wave V peak latency during the *cancalata*, *ekagrata* and *dharana* sessions but there was no change during the *dhyana* session.

In the literature, there is only one previous study of short latency auditory-evoked potentials in TM practitioners. In this study, at moderate stimulus intensities (40–50 dB), the wave V latency increased following meditation.[2] In contrast, at higher stimulus intensities, the wave V latency was slightly decreased by a comparison of the slopes and intercepts of stimulus intensity–latency functions. The authors suggested a possible effect of TM on brainstem activity. In the present study, there was no attempt to vary the stimulus intensity, which was kept at the 80 dB normal hearing level. This would fit in the category of a higher-intensity stimulus based on the categorization in the study.[2] In contrast to that study, even at this high-stimulus intensity, the latency of wave V did not decrease during either of the two meditation sessions (*dharana* and *dhyana*). In contrast, an increase in wave V peak latency was found in the *cancalata*, *ekagrata* and *dharana* sessions. No such increase was obtained in the *dhyana* session. An increase in the latency of an evoked potential component is taken to suggest that sensory information processing at the level of the underlying neural generator is delayed.[16] This suggests that in the *cancalata*, *ekagrata* and *dharana* mental states, sensory processing at the midbrain level was delayed. Another feature of the present study is that a difference is seen in the nature of the results in the two meditation sessions.

In the introduction, it was already mentioned that *dharana* and *dhyana* states have been described in an ancient yoga text, namely *Patanjali’s yoga sutras*. In this text, *dharana* literary means “fixing the mind on a specific object” (*Patanjali’s yoga sutras* Chapter 3 verse 1). The mind could be fixed on any point and. as long as disturbances from any corner are warded off, this mental state is called *dharana*. When *dharana* becomes effortless, it takes the form of *dhyana*, which is defined as the uninterrupted spontaneous flow of the mind toward the chosen object.

In contrast to this, the two control sessions, i.e. *cancalata* and *ekagrata* are described in another ancient text, the *Bhagavad Gita*.[17] The *cancalata* state is characterized by constant shifting of thoughts from one object to another. The *ekagrata* state is quite different from this and is similar to concentration. When haphazard thoughts are streamlined in a single direction, it is called *ekagrata*.

Hence, irrespective of whether meditators were in a state of random thinking (*cancalata*), channelized thought in concentration (*ekagrata*) or in a state of channelized thought as in meditation (*dharana*), there was a delay in sensory information processing, as mentioned above at the mid-brain (possibly the inferior colliculus) level. In contrast, when the mental state was characterized by a lack of effort in *dhyana*, no such change occurred.

Further studies are required to understand whether neural relay centers further along the auditory pathway would also change differently in *dharana* and *dhyana* states. The limitations of the present study are: (i) the fact that there was no attempt to vary stimulus intensities and hence the earlier findings of McEvoy, Frumkin and Harkins,[2] could not be examined, (ii) *ekagrata*, *dharana* and *cancalata* sessions were not different and cannot be ruled out as the VAS is essentially a subjective measure; no objective measure was taken. Only those subjects who achieved 75% of their ideal practice based of their subjective rating were included in the study. Again, the possibility that the sound stimulus influences all four practices cannot be ruled out. This is another limitation of the study.

Despite these limitations, the present study does demonstrate a difference between the *dharana* and *dhyana* states of meditation based on BAEP.[15,17,18]
